# Transdermal Nitroglycerin Delivery Using Acrylic Matrices: Design, Formulation, and *In Vitro* Characterization

**DOI:** 10.1155/2014/493245

**Published:** 2014-01-06

**Authors:** Houman Savoji, Amir Mehdizadeh, Ahmad Ramazani Saadat Abadi

**Affiliations:** ^1^Chemical and Petroleum Engineering Department, Sharif University of Technology, Azadi Avenue, Tehran 11365 8639, Iran; ^2^Hakim Pharmaceutical Co., 1370 Dr. Shariati Avenue, Gholhak, Tehran 11155 4465, Iran

## Abstract

Nitroglycerin (TNG) transdermal drug delivery systems (TDDSs) with different acrylic pressure-sensitive adhesives (PSAs) and chemical permeation enhancers (CPEs) were prepared. The effects of PSAs and CPEs types and concentrations on skin permeation and *in vitro* drug release from devices were evaluated using the dissolution method as well as the modified-jacketed Franz diffusion cells fitted with excised rat abdominal skin. It was demonstrated that the permeation rate or steady state flux (*J*_ss_) of the drug through the excised rat skin was dependent on the viscosity and type of acrylic PSA as well as the type of CPE. Among different acrylic PSAs, Duro-Tak 2516 and Duro-Tak 2054 showed the highest and Duro-Tak 2051 showed the lowest *J*_ss_. Among the various CPEs, propylene glycol and cetyl alcohol showed the highest and the lowest enhancement of the skin permeation of TNG, respectively. The adhesion properties of devices such as 180° peel strength and probe tack values were obtained. It was shown that increasing the concentration of CPE led to reduction in the adhesion property of PSA. Moreover, after optimization of the formulation, it was found that the use of 10% PG as a CPE and 25% nitroglycerin loading in Duro-Tak 2054 is an effective monolithic DIAP for the development of a transdermal therapeutic system for nitroglycerin.

## 1. Introduction

Nitroglycerin (1,2,3-propanetriol trinitrate) (TNG) is a vasodilator that has been widely used for the treatment of angina pectoris. For the acute treatment of angina, it is available as chewing capsules, sublingual tablets, and mouth spray. Injection solutions are available for the treatment of acute myocardial infarction. It decreases the smooth muscle tonus of blood vessels through the local production of nitrous oxide (NO). Because of its lipophilic nature, it is rapidly absorbed and extensively distributed when administrated orally, and afterwards it is excreted by the renal route. TNG is rapidly metabolized in the liver (half-life of 1 to 4 min) by hepatic enzymes to dinitrates and mononitrates and undergoes a massive hepatic first pass effect [1, 2]. Therefore, its bioavailability (BA) is below 1%. To bypass this first pass effect and improve the low bioavailability of TNG, the sublingual, intravenous, and transdermal administrations of TNG are possible solutions to overcome these problems. The sublingual bioavailability (38%) is still low, while the intravenous administration bioavailability is high enough, but it is an invasive procedure and it is mostly useful for the most acute infarction. Therefore, the transdermal administration of TNG is a possible solution to overcome these problems for long-term treatment of angina [1, 3].  Table 1 shows the comparison of three types of possible administration routs for TNG.

A transdermal route offers a number of advantages over the oral route, such as avoidance of gastrointestinal tract difficulties, avoidance of the first pass effect, being suitable when oral route is not desirable like vomiting, the capacity for multi-day therapy with a single application, quickly termination of administration by simple removal, and less chances of over- or under dosing [4, 5].

Transport of compounds via skin is considered to be a complex phenomenon, which allows the passage of certain chemicals into and across the skin. Among the various skin layers, stratum corneum (SC) is the rate-limiting barrier to percutaneous drug transport due to its desquamating “horny” properties [6, 7]. Due to the lipid-rich nature of the SC layer and its low water content, transport of hydrophilic or charged molecules across SC is low, while transport of lipophilic drug molecules such as TNG is higher due to their lipid miscibility with intercellular lipids around the cells in the SC layer [6, 8].

**Figure 1:**
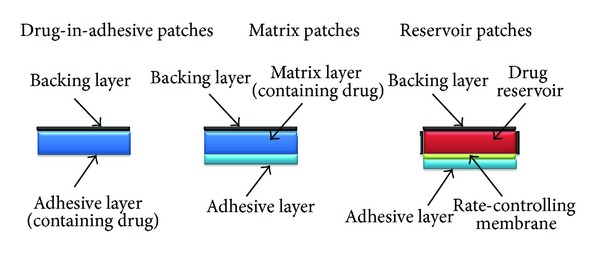
Characteristics and schematic of three different designs of TDDSs.

Preparation of transdermal drug delivery systems (TDDSs) consists of three basic designs: membrane control or reservoir patches (RPs), matrix or monolithic patches (MPs), and drug-in-adhesive-patches (DIAPs). Several factors should be considered before choosing an appropriate design for a particular compound: drug solubility, stability and release rate [4, 5]. When the characteristics of three different designs are compared, DIAPs are definitely simple in structure and more superior in terms of patient compliance and the commercial viewpoint. DIAP consists of a drug, additives, pressure-sensitive adhesive (PSA), backing film and release liner [9].  Table 2 and Figure 1 demonstrate characteristics and schematic of three different designs of TDDSs.

The first skin-contact adhesive application was the use of PSA in bandages for wound healing. These biocompatible PSAs mostly contain medical grade acrylic polymer with very low concentration of residue monomers which possess soft and constant adhesion and high cohesion level. They possess permeability of water vapor or air and minimal allergenic potential [10, 11]. Mehdizdeh et al. [12] studied the effects of various PSAs in a monolithic DIAP of fentanyl. According to their work, the permeation rate of fentanyl through the excised rat skin was dependent on the viscosity and type of acrylic PSA. Taghizadeh et al. [13] fabricated new fentanyl patch using acrylic/silicone PSA blends. They found that the effect of the ratio of silicone to acrylic PSA, polyvinyl pyrrolidone, and lauryl alcohol on the properties of a drug in adhesive patch containing fentanyl was different. In addition, they also prepared fentanyl patch with different functional and nonfunctional acrylic PSAs. Finally, they concluded that formulations with the highest percentage of –COOH functional PSA have displayed the lowest flux [14].

Since skin acts as a barrier to the delivery of most drugs, many chemical permeation enhancers (CPEs) have been used to enhance the transdermal penetration. A suitable CPE provides design flexibility with formulation chemistry and an easier possibility of patch application over a large area. CPEs can increase skin permeability by various mechanisms, including enhancing solubility, increasing partitioning into the SC (e.g., ethanol and propylene glycol), fluidizing the crystalline structure of SC, and causing dissolution of SC (e.g., oleic acid) [15, 16]. Qvist et al. [17] evaluated the release of eight CPEs from eight types of polymer adhesives using Franz diffusion cells. It was shown that all the enhancers were released completely from the adhesives and followed Higuchi law. The release rate was more dependent on the type of enhancer than on the type of polymers. Pichayakorn et al. [18] prepared novel nicotine transdermal patches using various CPEs. They reported that adding CPEs to polymer blends produced a faster release rate due to their greater hydrophilicity. Vávrová et al. [19] investigated feasibility of transdermal and dermal delivery of an antiviral. They showed that while dodecanol as CPE was ineffective, 1-dodecylazepan-2-one (Azone) and dodecyl 2-(dimethylamino) propionate (DDAIP) showed moderate activity. Mehdizadeh et al. [12] examined the effects of various CPEs in a transdermal patch of fentanyl. They demonstrated that type of CPE has a great influence on the permeation rate of fentanyl through the excised rat skin. They reported that among the various CPEs, propylene glycol and polyethylene glycol 400 showed the highest and the lowest enhancement ratios (ER) of the skin permeation of fentanyl, respectively. Oleic acid and cetyl alcohol moderately increased the skin permeation of fentanyl [12]. Nishida et al. [20] applied several surfactants as CPE to improve the penetration of valsartan in a monolithic drug-in-adhesive patch. They reported that a combination of isopropyl myristate (IPM)/diisooctyl sodium sulfosuccinate (AOT) most strongly enhanced the permeation of valsartan. Taghizadeh and Bajgholi [21] investigated a new liposomal-drug-in-adhesive patch for sodium diclofenac. They showed that liposomal-based nanocarrier as CPE encapsulates or associates sodium diclofenac.

In the present study, we focused on the design, formulation, and *in vitro* characterization of new drug-in-adhesive formulation of TNG transdermal patches using various pressure-sensitive adhesives (PSAs) and various chemical permeation enhancers (CPEs). The effects of PSAs and CPEs on skin permeation of TNG from DIAPs were evaluated using modified-jacketed Franz diffusion cells fitted with excised rat abdominal skin. Moreover, different formulations were evaluated with respect to adhesive properties such as peel strength and tack values. At the end, we aimed to design and optimize a home-made formulation of TNG which possesses similar characteristic of one of the existing DIA TNG patches.

## 2. Materials and Methods

Nitroglycerin was obtained by concentrating of nitroglycerin 1% (Merck, Germany) by evaporation to 18.2% nitroglycerin solution in ethanol. Acrylic pressure sensitive adhesives (PSAs): Duro-Tak 87-2054, and Duro-Tak 87-2051, Duro-Tak 87-2516, were provided by National Starch and Chemical Co., Bridgewater, NJ.. Oleic acid (OA), propylene glycol (PG), and cetyl alcohol (CA) as chemical permeation enhancers (CPEs) were purchased from INTERCHIMIE Co. (France).

Cotran 9720 polyethylene backing layers were donated by 3M. The backings have both low moisture vapor transmission rate and high oxygen transmission. These foster improved skin health by increasing moisture close to the skin to maintain skin hydration while allowing it to breathe.

Release liner, also known as peeling or protective liner, 3M Scotchpak 1022 liner, was donated by 3M.

Quadruple laboratory film applicator with a lateral guide plate and 4 thickness choices 90, 170, 250, and 500 *μ*m, and a 90 mm gap width was purchased from Sandberg & Schneidewind (Germany).

Support membrane (Spectra/Por 7 with cut off 14000 Daltons) to fix patches in the dissolution vessel was purchased from Spectrum (USA). All solvents and reagents used were of analytical reagent grade, and solutions were prepared with purified water (conductivity less than 1 *μ*S/cm). Reference nitroglycerin DIAP formulation was MinitranS 10 from 3M which releases 10 mg per 24 hr.

### 2.1. Preparation of DIAPs

DIAPs were prepared by using a quadruple laboratory film applicator with a lateral guide plate and four thickness choices, 90, 170, 250, and 500 *μ*m with 90 mm gap width (Sandberg & Schneidewind, Germany). The PSAs and CPEs used in the preparation of TNG DIAPs are listed in Table 3. The DIAPs were made of a flexible backing, a PSA containing TNG, and a release liner. The accurate amount of drug was weighed and thereafter CPE and PSA solution were added to the TNG solution and mixed with a magnetic stirrer (IKA, Germany) for 30 min. The concentration of each CPE was initially set according to Table 3. The amount of PSA in the formulations is about 70–80% and the amount of nitroglycerin is about 20–30% total weight. The mixture was then poured into the trough of the film applicator and spread on the Scotchpak 1022 release liner at a constant rate of about 1 m min^−1^ at a constant wet thickness of 500 *μ*m. The films were dried in ambient condition. The dried film with a thickness of about 150 *μ*m was laminated with Cotran 9720 backing. Prepared DIAPs were packed in opaque, white heat-sealed pouches. Round patches of 15 cm^2^ were die-cut and used for measurements.

### 2.2. Analysis of Nitroglycerin

Concentrations of nitroglycerin were measured by a validated HPLC method as follows. Nitroglycerin content was analyzed using HPLC UV (series 486 Waters, USA) at a detection wavelength of 220 nm. The column type was a reversed phase bond pack C18 (300 × 3.9 mm i.d., 10 *μ*m particle size, Waters). The mobile phase was 70 : 30 of methanol: water solution. The retention time and flow rate were approximately 6 minutes and 1 mL min^−1^, respectively.

### 2.3. *In Vitro* Release of Nitroglycerin

Determination of the nitroglycerin release pattern in different DIAPs and their comparison with reference patch were carried out using a USP 27 apparatus 5, paddle over disk, operating at 50 rpm in distillated water equilibrated to 32 ± 0.5°C. Due to adequate solubility of nitroglycerin corresponding to its concentration and provision of sink condition, 500 mL of distilled water was used as dissolution medium. One patch was applied flat on the disk with the release surface facing up (effective area available to diffusion was 15 cm^2^) and a support membrane on top of it. This membrane was dehydrated by immersion in purified water 1 h before application. At predetermined time intervals, 5 mL samples were collected and immediately replenished with fresh medium. The samples were analyzed for their nitroglycerin content using the validated HPLC method [22].

### 2.4. Rat Skin Permeation of Nitroglycerin

#### 2.4.1. Preparation of Rat Abdominal Skin

Male Sprague-Dawley rats (150–200 g) obtained from animal house of the Faculty of Pharmacy, University of Tehran, (Tehran, Iran) were sacrificed using diethyl ether asphyxiation. Hair of abdominal region was carefully removed and a 5 cm × 6 cm full-thickness skin was excised from this region from each sacrificed rat. Subcutaneous fat was carefully removed with scalpel. The excised rat skins were dipped and soaked in normal saline solution at ambient temperature and transferred for *in vitro* skin permeation study within 3-4 hours. Permission for the experiment was given by the Ethics Committee of the Tehran University of Medical Sciences.

#### 2.4.2. *In Vitro* Rat Skin Permeation Studies

Permeation investigation was carried out using excised rat abdominal skin in a modified-jacketed Franz diffusion cell with 3.14 cm^2^ effective diffusion area. The receptor compartment of the diffusion cell was completely filled with 36 mL of normal saline solution (0.9% Nacl) as receiver medium. Excess water was removed from the surface of the skin by gently rubbing with lint-free tissue paper. The DIAP was adhered to the epidermal side of the rat skin with slight pressure and then mounted over the receptor compartment. The O-ring and donor chamber with pregreased flange were placed over the DIAP. Any air bubbles remained in the receptor compartment and below the skin was carefully removed by gently tilting the diffusion cell. The receptor medium was well stirred by a magnetic stirrer (IKA, Germany) and the temperature was maintained at 32 ± 0.5°C using a thermostatic water pump bath (Brookfield, TC-101, USA). To maintain sink condition throughout the experiments, at predetermined time intervals (1, 2, 4, 8, 12, and 24 h), the receptor medium was completely withdrawn from the receptor compartment and replaced with fresh normal saline. The concentration of nitroglycerin was determined at each sampling by a fullyvalidated HPLC method and consequently the cumulative amount of nitroglycerin was calculated.

### 2.5. Adhesion Properties

Several methods have been used to evaluate the adhesive properties of PSAs. According to American Standard Test Methods (ASTM), peel adhesion tests are commonly performed to determine the adhesion of transdermal patch.

#### 2.5.1. Peel Adhesion 180° Test

The adhesive strength of the DIAPs was determined using an adhesion/release apparatus (Cheminstruments, AR 1000, USA) and by applying the 180° peel test according to the American Standard Test Methods (ASTM). The objective of the 180° peel test is to determine the peel force, in Newton (N), needed to remove the TDDS from the release liner using a 180° peel angle at a constant peel rate of 15.2 cm min^−1^. DIAP samples were prepared as ribbons of 25 mm width and 305 mm length at ambient temperature and humidity [23, 24].

#### 2.5.2. Probe Tack Test

The test that was used for evaluating the adhesive properties of PSA was Probe Tack test by using Probe Tack apparatus (PT 559, USA). This test is based on ASTM D2979 using 0.5 cm diameter Teel probe, a two-second dwell time, and the removal rate of 0.5 cm/sec. The values reported are the maximum force in (N) to remove the probe from the adhesive surface [25].

### 2.6. Statistical Analysis

All experiments were repeated three times, and their mean values are presented with the corresponding standard deviations. Student's *t*-test was performed to find the significant difference, if any, in the permeation rate between different DIAPs.

## 3. Results and Discussion

### 3.1. *In Vitro* Release of Nitroglycerin

Developing a discriminating dissolution medium using a proper dissolution apparatus is of tremendous value for a drug release study in TDDSs. The volume, pH, surface tension, and viscosity of medium are the most important parameters to be considered [12]. Our analytical results showed that the solubility of nitroglycerin in water is 225 mg L^−1^. Therefore, water may provide the sink condition for nitroglycerin patches without any surface active agents. In these experiments, 500 mL water was selected as the discriminative dissolution medium since it created the sink condition.

Drug absorption into the skin, generally, occurs by passive diffusion [25]. The rate of drug transport across the SC follows Fick's law of diffusion:

(1) $$\begin{array}{c} J=\frac{dM}{Sdt}=\frac{D\Delta ck}{h}, \end{array}$$

where *dM*/*Sdt*  (*J*) is the steady-state flux across the stratum corneum, *D* is the diffusion coefficient or diffusivity of drug molecules, Δ*c* is the drug concentration gradient across SC layer, *K* is the partition coefficient of the drug between skin and formulation medium, and *h* is the SC thickness [25]. In other words, the rate of drug transport does not depends only on its aqueous solubility, but is also directly proportional to its oil/water partition coefficient, its concentration in the formulation vehicle, and the surface area of the skin to which it is exposed; it is inversely proportional to the SC thickness.

In this study, different experimental transdermal formulations of 15 cm^2^ surface area were tested. The composition of the test formulations varied in the types of pressure sensitive adhesives and chemical permeation enhancers. The thickness of the transdermal patches was held constant. Test formulations were always compared to the reference patch, MinitranS 10 (3M).

**Figure 2:**
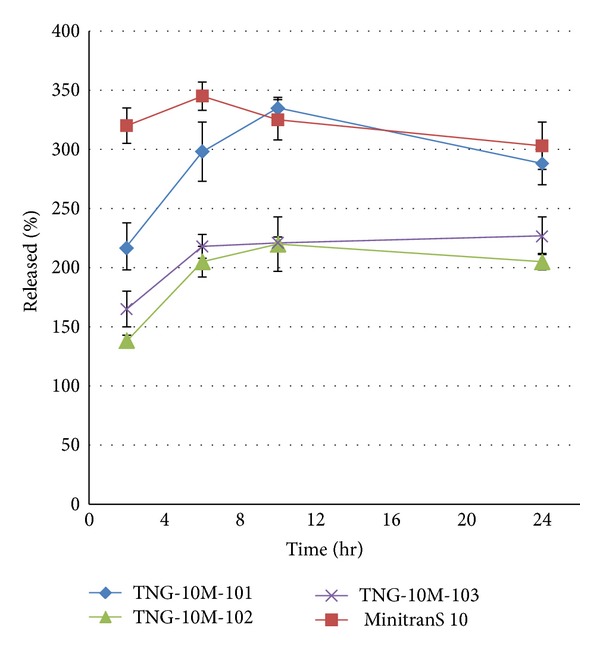
Comparative release of nitroglycerine from DIAP formulations with different PSAs and reference DIAP. Each value represents three tests.

The *in vitro* release rate profiles of nitroglycerin from various patches (TNG10-M10-101, TNG-10 M-102, TNG-10 M-103) with different pressure sensitive adhesives (Duro-Tak 2054, 2051, 2516) are shown in Figure 2. The amount of nitroglycerin released, as a percentage of the labeled amount of the dose absorbed *in vivo* are reported at different time points in this Figure. The results show a very fast initial burst release for all formulations tested. 80–90% of drug was detected in dissolution medium within 6 hr. The comparative release profiles of different DIAPs showed that the DIAP formulation TNG-10 M-101 which contains PSA Duro-Tak 2054 had the similar profile to reference patch MinitranS 10. Therefore, for the study of the effect of CPEs, we choose Duro-Tak 2054 as PSA.

**Figure 3:**
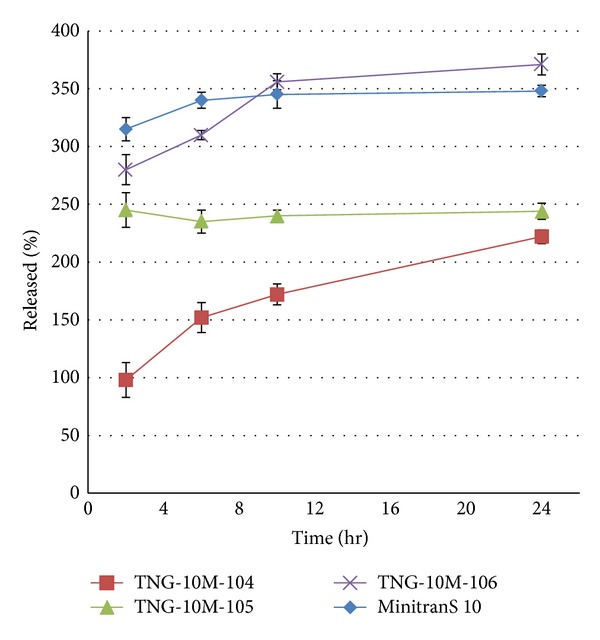
The effect of CPEs on release profile of nitroglycerin from DIAP formulation with Duro-Tak 2054. Each value represents three tests.

The effect of adding three chemical permeation enhancers such as PG, OA, and CA on release profile of DIAPs with PSA Duro-Tak 2054 was shown in Figure 3. As it is clear, the formulation containing PG has a similar release profile to the reference DIAP.

These results show that the main transfer resistance to diffusion of drugs is SC layer of the skin. Because in dissolution test all drugs were delivered out from the patches, and no mass transfer resistance exists in the absence of the skin. It is also obvious that the release into aqueous medium will mainly depend on the solubility of the drug in the receptor medium, and the results will be different from the trend of partitioning into the skin.

### 3.2. Rat Skin Permeation Parameters

#### 3.2.1. Influence of Various PSAs on Skin Permeation

**Figure 4:**
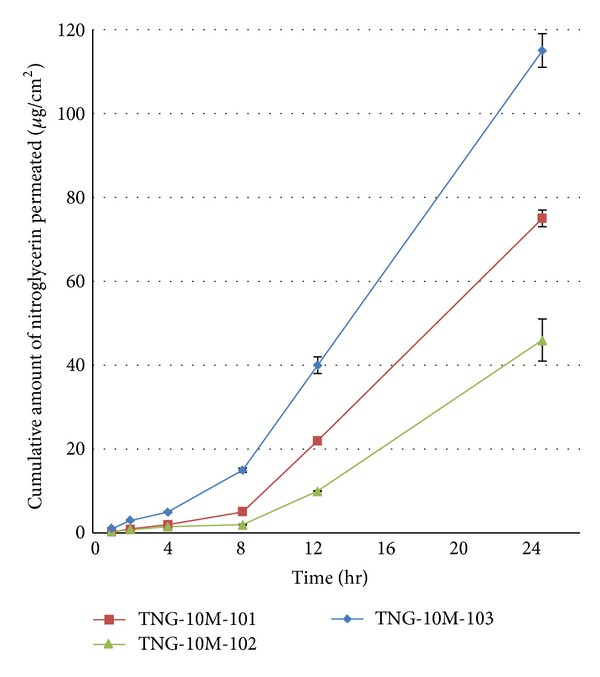
Permeation profiles of nitroglycerin through excised rat abdominal skin from different DIAPs prepared using various PSAs.

The skin permeation rate of nitroglycerin through excised rat abdominal skin from different DIAPs prepared with various acrylic PSAs were determined. The permeation profiles of nitroglycerin are shown in Figure 4. Permeation parameters, including the permeation rate or steady state flux (*J*_ss_) and lag time (*T*_*L*_), were calculated from the profiles and are presented in Table 4. The permeation rates of nitroglycerin from DIAPs through rat skin were in the range 2.81–6.25 *μ*g cm^−2^ h^−1^. Among four different PSAs used in this study, Duro-Tak 2516 resulted in the highest skin permeation rate of nitroglycerin (6.25 *μ*g cm^−2^ h^−1^), while Duro-Tak 2051 showed the lowest permeation rate (2.81 *μ*g cm^−2^ h^−1^). These results may be explained by the viscosity and the amount of solid content. According to manufacturer's leaflet, the viscosities of Duro-Tak 2051 and Duro-Tak 2516 are 4000 and 4350 mPa·s and the solids content of 51% and 41.4%, respectively. Also, there were significant differences in the lag time between the PSAs, ranging from 5.6 to 7.8 h (*P* < 0.05). Subsequently, Duro-Tak 2516 and Duro-Tak 2054 were used in further development of the nitroglycerin DIAP, because they revealed the highest steady state flux and the shortest lag time, which are essential for an appropriate therapeutical effect of nitroglycerin.

#### 3.2.2. Influence of CPEs on Skin Permeation

**Figure 5:**
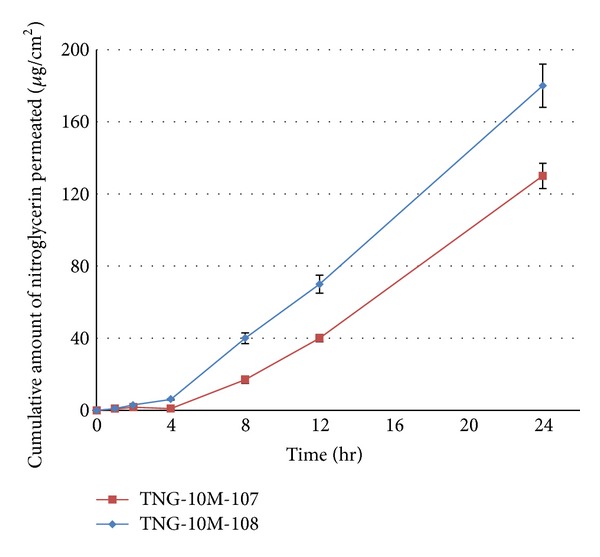
Permeation profiles of nitroglycerin through excised rat skin from different DIAPs prepared using Duro-Tak 2516 and various CPEs.

Incorporation of CPEs in the DIAP was essential for increasing the permeation rate of nitroglycerin from the patches. In this study, one lipophilic CPE and one hydrophilic CPE were selected and their effects on the permeation of nitroglycerin from DIAPs (TNG-10M-107, TNG-10M-108) prepared with Duro-Tak 2516 (showed the highest permeation rate in previous study) through excised rat skin were investigated at a preliminary concentration of 10%. The skin permeation profiles of the drug from the DIAPs are shown in Figure 5. The permeation parameters calculated from the obtained profiles are presented in Table 5.

Among the two CPEs used, PG showed the highest enhancing effect for nitroglycerin. The mechanism of action of various CPEs may be attributed to their activity on lipophilic matrix and/or hydrophilic protein gel in stratum corneum [4, 5]. CPEs act through interaction with intercellular lipids, leading to disruption of their organization and increasing their fluidity. Some of them also interact with intercellular protein, keratin denaturation (e.g., oleic acid), while PG acts by both mechanisms [4]. These last enhancement mechanisms are consistent with the findings obtained in our experiments. On the other hand, OA moderately increased the skin permeation of nitroglycerin.

Incorporation of PG into the acrylic PSA matrix significantly enhanced the permeation rate and shortened the *T*_*L*_ of nitroglycerin. In conclusion, the maximum *J*_ss_ obtained from DIAP prepared with Duro-Tak 2516 and 10% PG was 8.85 *μ*g cm^−2^ h^−1^. Among these CPEs, the lag time was predominately reduced by two enhancers, OA and PG. Thus, they can cause disruption of the lipid matrix of stratum corneum and faster penetration of nitroglycerin through skin.

It should be noted that the excessive or inappropriate usage of CPEs may lead to loss of the PSA's mechanical properties [12] (discussed in the next section).

These permeation results are still far from the permeation rate of MinitranS 10 (control). None of these formulations are able to release 10 mg per 24 hr. Therefore, in the next section, optimization of the formulations has been done by reducing the amount of PSAs.

#### 3.2.3. Optimization of the Formulation

In order to improve the cumulative amount of TNG released in our formulations and meet the corresponding amount for the control patch (MinitranS 10) (10 mg per 24 hr), the amount of PSAs were decreased in the formulations (Table 3). In this set of experiment, two PSAs (Duro-Tak 2516, Duro-Tak 2054) which had the highest permeation rate were selected. PG was chosen as the appropriate CPE considering its ability to enhance the permeation rate compared to the others.

**Figure 6:**
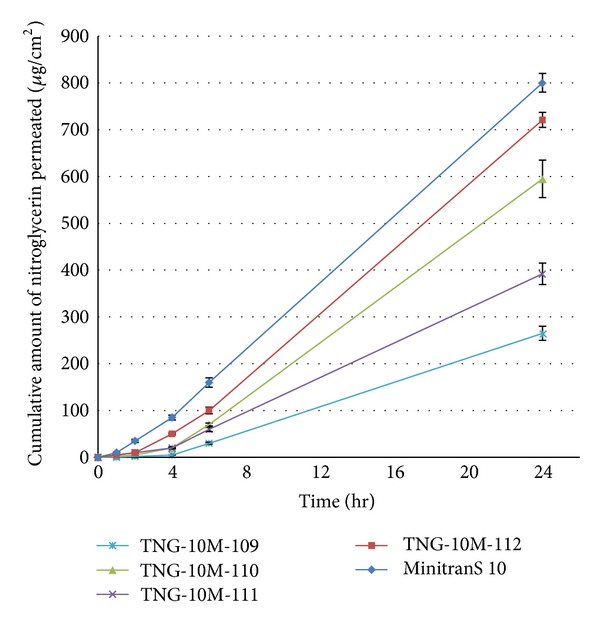
Permeation profiles of nitroglycerin through excised rat skin from different optimized formulations.

Figure 6 and Table 6 show the skin permeation profiles and the permeation parameters calculated from the obtained profiles of the TNG from the optimized DIAPs, respectively.

The permeation rates of nitroglycerin from optimized DIAPs through rat skin show that the formulation containing Duro-Tak 2054 as PSA and PG as CPE has the most similar profile with control patch (MinitranS 10). According to Table 6, it releases 33.12 *μ*g cm^−2^ h^−1^ with a lag time of 2.3 hr which corresponds to approximately 10 mg per day MinitranS 10. Although, formulation containing Duro-Tak 2516 and PG also releases 28.91 *μ*g cm^−2^ h^−1^ (corresponds to 10 mg per day MinitranS 10), but it shows higher lag time which is far from the control patch. This means that we have designed a home-made drug-in-adhesive transdermal patch of nitroglycerin which has the most similar *in vitro* characteristics to the commercial one.

#### 3.2.4. Peel Adhesion 180° Test and Probe Tack Test

Eight different formulations were prepared as described above. The results (average load) of the effects of types and concentrations of two different chemical permeation enhancers from different chemical categories on adhesion properties of PSA system are presented in Table 7. The results of types of CPEs on Probe TACK test are depicted in Table 8.

For both test methods, it was found that the presence of penetration enhancer affected peel force, while the type of the enhancer also did. The inclusion of penetration enhancers in matrix with PSAs often affects the mechanical properties of the system, because enhancers act like plasticizers. Figure 5 shows that the peel force decreased when the concentration of two enhancers increased from 0% to 15%. Maximum peel force was obtained with Duro-Tak 87-2054 and 0% concentration of enhancers. Results in Figure 5 and Tables 3 and 4 show that both enhancers have negative effects on mechanical properties of systems, but negative effects for oleic acid are more significant than PG.

## 4. Conclusions

### 4.1. *In Vitro* Release of Nitroglycerin

The aim of this study was to “design and *in vitro* evaluate transdermal drug delivery system for Nitroglycerin” for the treatment of angina pectoris. Drug-in-adhesive patches were prepared and evaluated by dissolution method. It was concluded that the release profile of nitroglycerin depends on viscosity and type of pressure sensitive adhesive and type and concentration of selected chemical permeation enhancer.

### 4.2. Rat Skin Permeation

The permeation rate of nitroglycerin in Duro-Tak 2516 was the highest among the three PSAs examined. It was clearly shown that PG showed the highest steady state flux among three CPEs tested. On the other hand, increasing the concentration of CPEs led to improving steady state flux of the drug (*J*_ss_) but decreased the adhesion strength. This phenomena may be explained by excessive plasticizing of PSA and result in poor adhesion [4]. This work showed that these PSAs that are commonly used in TDDS are not tolerated to enhancers. In summary, the results of the study indicated that the use of a 10% PG as a CPE and 25% nitroglycerin loading in Duro-Tak 2054 is an effective monolithic DIAP for the development of a transdermal therapeutic system for nitroglycerin.

### 4.3. Peel Adhesion

The results of the 180° peel test and Probe Tack test showed that concentrations of enhancer affected the adhesive properties DIAP. Since the release profile of drug is influenced by type and concentration of enhancer; therefore, these factors should be considered and optimized during the developmental phase of TDDS.

## Figures and Tables

**Table 1:** Characteristics of three types of possible administration routs for NG.

	Sublingual tablets	Infusion	Transdermal Patch
Therapeutic effect (min)	1–3	1-2	30–60
Duration of action (hr)	0.5–1	0.05–0.1	8–10
Bioavailability (%)	38.5	<100	75
Peak plasma concentration (ng/mL)	3	—	<0.5

**Table 2:** Characteristics of DIAPs and MPs versus RPs.

Characteristics	Drug in adhesive or matrix patch	Reservoir patch
Structure	Simple thin layer	Complex multilayer
Formulation	Complex	Simple
Skin conformability	Good	Some discomfort
Size adjustment	Easy	Difficult
Dose dumping	Low potential	Possible break of rate controlling layer

**Table 3:** Various transdermal DIAPs nitroglycerin formulations.

Compound	TNG-10M-101	TNG-10M-102	TNG-10M-103	TNG-10M-104	TNG-10M-105	TNG-10M-106	TNG-10M-107	TNG-10M-108	TNG-10M-109	TNG-10M-110	TNG-10M-111	TNG-10M-112
Nitroglycerin (mL)	4	4	4	3.9	3.9	3.9	3.9	3.9	2.6	2.6	2.6	2.6
Duro-Tak 2054 (gr)	5.4	—	—	4.7	4.7	4.7	—	—	—	—	2.5	2.5
Duro-Tak 2051 (gr)	—	5.4	—	—	—	—	—	—	—	—	—	—
Duro-Tak 2516 (gr)	—	—	5.4	—	—	—	4.7	4.7	2.5	2.5	—	—
Cetyl Alcohol (gr)	—	—	—	0.3	—	—	—	—	—	—	—	—
Oleic Acid (gr)	—	—	—	—	0.3	—	0.3	—	—	—	—	—
PG (gr)	—	—	—	—	—	0.3	—	0.3	—	0.19	—	0.19

**Table 4:** Permeation parameters of TNG through excised rat skin from drug-in-adhesive patches prepared using various acrylic pressure sensitive adhesives.

Permeation parameters		
DIAP	*J*_ss_ (*μ*g cm^−2^ h^−1^)	*T*_L_ (h)
Duro-Tak 2516 (TNG-10M-103)	6.25 ± 0.11	5.6 ± 0.4
Duro-Tak 2054 (TNG-10M-101)	4.38 ± 0.23	6.9 ± 0.3
Duro-Tak 2051 (TNG-10M-102)	2.81 ± 0.16	7.8 ± 0.4

**Table 5:** Permeation parameters of two formulations with different chemical permeation enhancers.

Permeation parameters		
DIAP	*J*_ss_ (*μ*g cm^−2^ h^−1^)	*T*_L_ (h)
TNG-10M-108 (PG)	8.85 ± 0.41	3.7 ± 0.5
TNG-10M-108 (OA)	6.62 ± 0.64	4.9 ± 0.3

**Table 6:** Permeation parameters of TNG through excised rat skin from optimized DIAPs.

Permeation parameters		
DIAP	*J*_ss_ (*μ*g cm^−2^ h^−1^)	*T*_L_ (h)
MinitranS 10	34.84 ± 0.45	1.2 ± 0.2
TNG-10M-112	33.12 ± 0.54	2.3 ± 0.3
TNG-10M-110	28.91 ± 0.22	2.8 ± 0.5
TNG-10M-111	18.84 ± 0.32	2.9 ± 0.5
TNG-10M-109	13.02 ± 0.19	3.6 ± 0.3

**Table 7:** Effect of type and concentration of CPEs on peel force of PSA Duro-Tak 2054.

Percent of CPE (%)	Peel Force (N)	
	Oleic Acid	PG
0	17.2 ± 1.6	17.2 ± 1.6
5	4.9 ± 0.7	14.9 ± 1.4
10	3.6 ± 0.4	14.1 ± 0.8
15	2.3 ± 0.5	13.3 ± 0.6

**Table 8:** Effect of types of CPEs on TACK test.

Formulation	Tack Force (N)
Duro-Tak 2054	6.35 ± 0.54
Duro-Tak 2054 and 10% PG	4.78 ± 0.86
Duro-Tak 2054 and 10% Oleic Acid	3.03 ± 0.65

## References

[1] Yu DK, Williams RL, Benet LZ, Lin ET, Giesing DH (1988). Pharmacokinetics of nitroglycerin and metabolites in humans following oral dosing. *Biopharmaceutics and Drug Disposition*.

[2] Hashimoto S, Kobayashi A (2003). Clinical pharmacokinetics and pharmacodynamics of glyceryl trinitrate and its metabolites. *Clinical Pharmacokinetics*.

[3] Sun JX, Piraino AJ, Morgan JM (1995). Comparative pharmacokinetics and bioavailability of nitroglycerin and its metabolites from transderm-nitro, nitrodisc, and Nitro-Dur II systems using a stable-isotope technique. *The Journal of Clinical Pharmacology*.

[4] Prausnitz MR, Mitragotri S, Langer R (2004). Current status and future potential of transdermal drug delivery. *Nature Reviews Drug Discovery*.

[5] Prausnitz MR, Langer R (2008). Transdermal drug delivery. *Nature Biotechnology*.

[6] Oshizaka T, Todo H, Sugibayashi K (2012). Effect of direction (epidermis-to-dermis and dermis-to-epidermis) on the permeation of several chemical compounds through full-thickness skin and stripped skin. *Pharmaceutical Research*.

[7] Stamatialis DF (2007). *Drug Delivery Through Skin: Overcoming the Ultimate Biological Membrane. Membranes for the Life Sciences*.

[8] Anissimov YG, Jepps OG, Dancik Y, Roberts MS (2013). Mathematical and pharmacokinetic modelling of epidermal and dermal transport processes. *Advanced Drug Delivery Reviews*.

[9] Guy RH, Schäfer-Korting M (2010). Transdermal drug delivery drug delivery. *Handbook of Experimental Pharmacology*.

[10] Czech Z, Kurzawa R (2007). Acrylic pressure-sensitive adhesive for transdermal drug delivery systems. *Journal of Applied Polymer Science*.

[11] Tan HS, Pfister WR (1999). Pressure-sensitive adhesives for transdermal drug delivery systems. *Pharmaceutical Science and Technology Today*.

[12] Mehdizadeh A, Ghahremani MH, Rouini MR, Toliyat T (2006). Effects of pressure sensitive adhesives and chemical permeation enhancers on the permeability of fentanyl through excised rat skin. *Acta Pharmaceutica*.

[13] Taghizadeh SM, Soroushnia A, Mirzadeh H, Barikani M (2009). Preparation and in vitro evaluation of a new fentanyl patch based on acrylic/silicone pressure-sensitive adhesive blends. *Drug Development and Industrial Pharmacy*.

[14] Taghizadeh SM, Soroushnia A, Mohamadnia F (2010). Preparation and in vitro evaluation of a new fentanyl patch based on functional and non-functional pressure sensitive adhesives. *AAPS PharmSciTech*.

[15] Karande P, Jain A, Ergun K, Kispersky V, Mitragotri S (2005). Design principles of chemical penetration enhancers for transdermal drug delivery. *Proceedings of the National Academy of Sciences of the United States of America*.

[16] Zbytovská J, Vávrová K, Kiselev MA, Lessieur P, Wartewig S, Neubert RHH (2009). The effects of transdermal permeation enhancers on thermotropic phase behaviour of a stratum corneum lipid model. *Colloids and Surfaces A*.

[17] Qvist MH, Hoeck U, Kreilgaard B, Madsen F, Frokjaer S (2002). Release of chemical permeation enhancers from drug-in-adhesive transdermal patches. *International Journal of Pharmaceutics*.

[18] Pichayakorn W, Suksaeree J, Boonme P, Amnuaikit T, Taweepreda W, Ritthidej GC (2012). Nicotine transdermal patches using polymeric natural rubber as the matrix controlling system: effect of polymer and plasticizer blends. *Journal of Membrane Science*.

[19] Vávrová K, Lorencová K, Klimentová J, Novotný J, Holý A, Hrabálek A (2008). Transdermal and dermal delivery of adefovir: effects of pH and permeation enhancers. *European Journal of Pharmaceutics and Biopharmaceutics*.

[20] Nishida N, Taniyama K, Sawabe T, Manome Y (2010). Development and evaluation of a monolithic drug-in-adhesive patch for valsartan. *International Journal of Pharmaceutics*.

[21] Taghizadeh SM, Bajgholi S (2011). A new liposomal-drug-in-adhesive patch for transdermal delivery of sodium diclofenac. *Journal of Biomaterials and Nanobiotechnology*.

[22] Shen J, Burgess DJ (2012). Accelerated in-vitro release testing methods for extended-release parenteral dosage forms. *Journal of Pharmacy and Pharmacology*.

[23] da Silva LFM, Öchsner A, Adams RD (2011). *Handbook of Adhesion Technology*.

[24] Satas D (1982). *Handbook of Pressure-Sensitive Adhesive Technology*.

[25] Puglia C, Bonina F, Trapani G, Franco M, Ricci M (2001). Evaluation of in vitro percutaneous absorption of lorazepam and clonazepam from hydro-alcoholic gel formulations. *International Journal of Pharmaceutics*.

